# RETRACTION: The Impact of Ulinastatin on Wound Infection and Healing in Patients With Burn Wounds: A Meta‐Analysis

**DOI:** 10.1111/iwj.70282

**Published:** 2025-03-03

## Abstract

**RETRACTION:** WangP.‐J. , QinG.‐J. , ShuY.‐Z. , and ZhangW.‐N. , “The Impact of Ulinastatin on Wound Infection and Healing in Patients With Burn Wounds: A Meta‐Analysis,” International Wound Journal 21, no. 4 (2024): e14562, 10.1111/iwj.14562.38130102 PMC10957368

The above article, published online on 21 December 2023, in Wiley Online Library (http://onlinelibrary.wiley.com/), has been retracted by agreement between the journal Editor in Chief, Professor Keith Harding; and John Wiley & Sons Ltd. Following an investigation by the publisher, all parties have concluded that this article was accepted solely on the basis of a compromised peer review process. The editors have therefore decided to retract the article. The authors did not respond to our notice regarding the retraction.

**RETRACTION:** P.‐J. Wang , G.‐J. Qin , Y.‐Z. Shu , and W.‐N. Zhang , “The Impact of Ulinastatin on Wound Infection and Healing in Patients With Burn Wounds: A Meta‐Analysis,” International Wound Journal 21, no. 4 (2024): e14562, 10.1111/iwj.14562.38130102 PMC10957368

The above article, published online on 21 December 2023, in Wiley Online Library (http://onlinelibrary.wiley.com/), has been retracted by agreement between the journal Editor in Chief, Professor Keith Harding; and John Wiley & Sons Ltd. Following an investigation by the publisher, all parties have concluded that this article was accepted solely on the basis of a compromised peer review process. The editors have therefore decided to retract the article. The authors did not respond to our notice regarding the retraction.

